# Distribution and Source of Polycyclic Aromatic Hydrocarbons (PAHs) in Water Dissolved Phase, Suspended Particulate Matter and Sediment from Weihe River in Northwest China

**DOI:** 10.3390/ijerph121114148

**Published:** 2015-11-06

**Authors:** Yuyun Chen, Rui Jia, Shengke Yang

**Affiliations:** 1 Key Laboratory of Subsurface Hydrology and Ecology in Arid Areas (Ministry of Education), Chang’an University, Xi’an 710054, China; 2 School of Environmental Science and Engineering, Chang’an University, Xi’an 710054, China; E-Mails: chdjr0119@163.com (R.J.); ysk110@126.com (S.Y.)

**Keywords:** ecological risk assessment, polycyclic aromatic hydrocarbons (PAHs), sources evaluation, Weihe River

## Abstract

Weihe River is a typical river located in the arid and semi-arid regions of Northwest China. In this study, the distribution and sources of 16 polycyclic aromatic hydrocarbons (PAHs) in Weihe River were investigated. The concentrations of ∑PAHs ranged from 351 to 4427 ng/L with a mean value of 835.4 ng/L in water dissolved phase (WDP), from 3557 ng/L to 147,907 ng/L with a mean value of 20,780 ng /L in suspended particulate matter (SPM), and from 362 to 15,667 ng/g dry weight (dw) with a mean value of 2000 ng/g dw in sediment, respectively. The concentrations of PAHs in Weihe River were higher compared with other rivers in the world. In both WDP and sediment, the highest concentrations of ∑PAHs were observed in the middle reach, while the lowest concentrations of ∑PAHs were found in the lower reach. For SPM, however, the PAHs concentrations in the lower reach were highest and the PAHs concentrations in the upper reach were lowest. The ratios of anthracene/(anthracene + phenanthrene) and fluoranthene/(fluoranthene + pyrene) reflected a pattern of both pyrolytic and petrogenic input of PAHs in Weihe River. The potential ecosystem risk assessment indicated that harmful biological impairments occur frequently in Weihe River.

## 1. Introduction

Polycyclic aromatic hydrocarbons (PAHs) are a class of diverse organic compounds containing two or more fused aromatic rings of carbon and hydrogen atoms. PAHs are ubiquitous contaminants in the environment. These compounds are generally generated by natural and anthropogenic processes and can be introduced into the environment through various routes. PAHs are of environmental concern due to their toxic, mutagenic and carcinogenic potential [1]. Due to their environmental concern, 16 PAHs were designated as priority pollutants by the United States Environmental protection Agency (US EPA) and seven PAHs were designated as potentially carcinogenic pollutants by the US EPA. For these reasons, PAHs’ behavior, transport, fate and environmental risk to ecological systems have become an advanced research hotspot in environmental fields all over the world [2,3,4,5,6,7,8,9].

A lot of papers have reported on PAHs in rivers in China [3,4,6,7,8,9,10,11,12,13]. However, almost all of these rivers were located in the humid and semi-humid regions in North, East, South or Northeast China. There are also many rivers located in the arid and semi-arid region of Northwest China, but no study about PAHs in these rivers has been published. Weihe River is a typical river located in the arid and semi-arid region in Northwest China. This basin is the most developed area in Northwest China and is under rapid industrialization and urbanization. China is now planning to carry out its “One Belt and One Road” program. The Weihe River basin is in an important position in the Silk Road economic belt. Xi’an City, the national cooperation platform of “One Belt and One Road”, locates in the Weihe River basin. Only the car ownership of Xi’an City is more than 1,300,000. More than 2.9 million people were living in the Weihe River basin in 2007. A lot of energy and chemical enterprises are located in this area. A large volume of raw sewage drains off into Weihe River or its branches, which inevitably results in bad water quality. With the rapid increase in population and economic wealth, the insufficient water supply and degradation of water environment have become the most restrictive factors for the basin’s economic development. The immense pressure on the area’s ecosystem makes the study of Weihe River necessary to safeguard its ecosystem health, so it is very important to study the characterization and distribution of PAHs in Weihe River.

The objectives of the present work were to determine the levels and spatial distribution of PAHs in Weihe River, and to identify the major sources of PAHs in this area. The results would be used to provide data for comparison with other rivers and to assess potential ecotoxicological effect.

## 2. Materials and Methods

### 2.1. Chemicals and Instruments

The 16 polycyclic aromatic hydrocarbons (PAHs) employed in this research were naphthalene (NAP), acenaphthene (ACE), acenaphthylene (ACY), fluorene (FLU), phenanthrene (PHE), anthracene (ANT), fluoranthene (FLU), pyrene (PYR), benzo[a]anthracene (BaA), chrysene (CHR), benzo[b]fluoranthene (BbF), benzo[k]fluoranthene (BkF), benzo[a]pyrene (BaP), dibenzo[a,h]anthracene (DhA), benzo[g,h,i]perylene (BgP) and indeno[1,2,3-c,d]pyrene (IcP) which were purchased from AccuStandard, Inc. (New Haven, KY, USA). All solvents used were HPLC grade or equivalent.

The PAHs were analyzed using an Agilent 6890A gas chromatography−5975C ion trap mass spectrometry (GC/MS) system equipped with a DB-EUPAH(20 m × 0.18 mm × 0.14 μm) capillary column (Agilent Technologies, Palo Alto, CA, USA). The carrier gas was high-purity helium (He). The injection port temperature was maintained at 270 °C. The GC-MS temperature program started at 60 °C for 1 min and then increased at a rate of 11 °C per minute to 270 °C. The temperature was then increased to 300 °C with a rate of 1.5 °C per minute and held at 300 °C for 2 min. The analysis was performed using the selected ion monitoring (SIM) mode with a splitless injection. The determination and quantification of 16 PAHs in the samples was achieved based on matching the compound ionization and retention time with those of the standard mixture of PAHs. The RSDs of retention time and peak area for the injection of 16 PAHs were all below than 0.5% and 2%, respectively.

### 2.2. Study Area and Sample Collection

**Figure 1:**
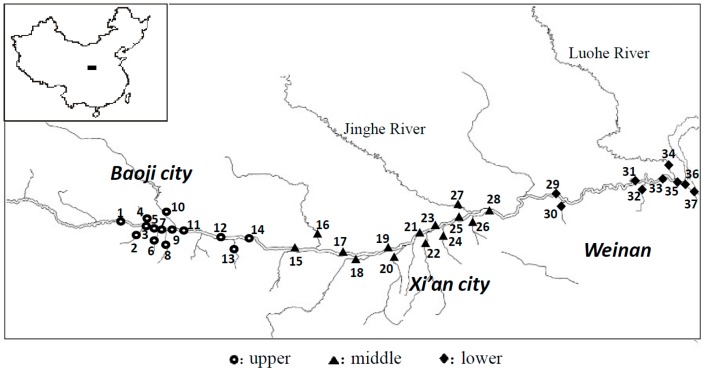
The study area and sampling locations in the Weihe River, China.

The sampling sites are illustrated in Figure 1. A total of 37 sampling sites along Weihe River and its tributaries were selected. Among the 37 sampling sites, 16 sites locate in its 16 main tributaries and 21 sites locate in the main stream of Weihe River. Fourteen, fourteen and nine sites were located in the upper (sites 1–14), middle (sites 15–28) and lower (sites 29–37) reaches of Weihe River, respectively. A total of 73 samples, including 37 water samples and 36 sediment samples were collected from the 29–31 August, 2014. On 31 August, the Weihe river flow at site 35 was 245 m^3^/s. The suspended particulate matter (SPM) samples are derived from water dissolved phase (WDP) in the laboratory. Water samples were collected from 0.5 m below the water surface using 2.5 L glass jars. The surface sediment samples were collected using grab sampler. During the whole sampling process global position system (GPS) was used to locate the sampling stations.

In each sampling point, 2.5 L of water (one amber bottle) were collected and transported to the laboratory. The separation of WDP and SPM was achieved by filtration according to the method of Montuori and Triassi [5]. Water samples were filtered through a previously kiln-fired (400 °C overnight) GF/F glass fiber filter (47 mm × 0.7 μm; Whatman, Maidstone, UK). Filters (SPM) were kept in the dark at −20 °C until analysis. Dissolved phase refers to the fraction of contaminants passing through the filter. This includes the compounds that are both truly dissolved as well as those associated with colloidal organic matter. These filtrates were kept in the dark at 4 °C and extracted within 24 h. Surface sediment (0–20 cm) samples were collected by using a grab sampler (Van Veen Bodemhappe 2 L, Kiel, Germany) and put in aluminum containers. An adequate quantity of surface sediment was collected in each sampling point. All samples were transported to the laboratory directly after sampling and kept at 4 °C before analysis.

### 2.3. PAHs Extraction

#### 2.3.1. Water Dissolved Phase

Solid phase extraction (SPE) cartridges system from Supelco (Sigma–Aldrich Corp., Saint Louis, MO, USA) was used to extract water dissolved phase samples according to the method of Chen *et al.* [9]. Before the extraction, the C_18_-bonded phase containing 500 mg of reversed phase octadecyl (Supelclean ENVI-18, Sigma–Aldrich Corp.) was first washed with 5 mL of dichloromethane, 5 mL methanol and5 mL ultra-pure water, respectively. About 50 mL methanol was added to the water sample (1 L) in order to improve the recovery. Then the solution was percolated through the cartridges with a flow rate of 3 mL·min^−1^ under vacuum pump. After extraction, the PAHs trapped were eluted to a glass tube by 5 mL dichloromethane. Two g anhydrous Na_2_SO_4_ was used to remove trace amount of water. The solvent fractions were then evaporated on a rotary evaporator, and exchanged by hexane to a final volume of 1 mL.

#### 2.3.2. Suspended Particulate Phase

The SPM content was determined by gravimetry, after drying the glass fiber filter in an air-heated oven (55 °C until constant weight) and equilibrated at room temperature in a desiccator. Suspended particulate phase containing glass fiber filters were cut into pieces and spiked with the surrogate (10 ng of anthracene-d10, pyrene-d10 and perylene-d12), and then extracted three times by ultrasonic-assisted solvent extraction in 20 mL of dichloromethane for 1 h followed by centrifugation. Then 10 mL of supernatant was filtered through a silica gel column (3 g) with 11 mL 1:1 (v/v) elution of hexane and dichloromethane. Anhydrous Na_2_SO_4_ (0.5 g) was used to remove moisture. The solvent fractions were then evaporated on a rotary evaporator, and exchanged by hexane with a final volume of 0.5 mL.

#### 2.3.3. Sediment

The PAHs in sediment were extracted by ultrasonic-assisted solvent extraction according to the method by Chen *et al.* [9]. The dry sediments were carefully collected, homogenized and passed through 250 μm standard sieve. Sample preparation included homogeneous mixing of 2 g of sediment sample with 0.5 g anhydrous Na_2_SO_4_ to remove moisture and ultrasonication in 20 mL of dichloromethane for 1 h followed by centrifugation. Then 10 mL of supernatant was filtered through 3 g of silica gel column with 11 mL 1:1 (v/v) elution of hexane and dichloromethane. The solvent fractions were then evaporated on a rotary evaporator, and exchanged with hexane with a final volume of 1 mL.

### 2.4. Parameters Determination

The total organic carbon (TOC) of sediment was determined by TOC analyzer (TOC-VCPH; Shimadzu Corp., Shimadzu, Japan). Chemical oxygen demands (COD) of water samples were determined by COD determinator (Shanghai; China). Electric conductivity (EC); total dissolved solids (TDS) and salinity were determined by a HQ30D water quality analyzer (Shanghai; China). The pH value was determined by a pH meter (Mettler Toledo, Columbus, OH, USA).

### 2.5. Quality Assurance

For every set of samples, a procedural blank and spike sample consisting of all reagents was run to check for interference and cross contamination. The method detection limits (MDLs) of PAHs were calculated as three times the standard deviation of the PAHs level in procedural blanks. A strict regime of quality control was employed before the onset of the sampling and analysis program. The surrogate averaged recoveries in the dissolved phase were 90.2% ± 3.9% for anthracene-d_10_, 94.6% ± 5.9% for pyrene-d_10_ and 95.1% ± 6.6% for perylene-d_12_. In the SPM samples, recoveries were 85.4% ± 6.4% for anthracene-d_10_, 92.1% ± 5.6% for pyrene-d_10_ and 97.8% ± 6.1% for perylene-d_12_. Finally, in the sediment samples the averaged recoveries were the following: 87.4% ± 6.8% for anthracene-d_10_, 92.2% ± 7.6% for pyrene-d_10_ and 101% ± 6.4% for perylene-d_12_.

## 3. Results

### 3.1. PAHs Concentrations in WDP

**Figure 2:**
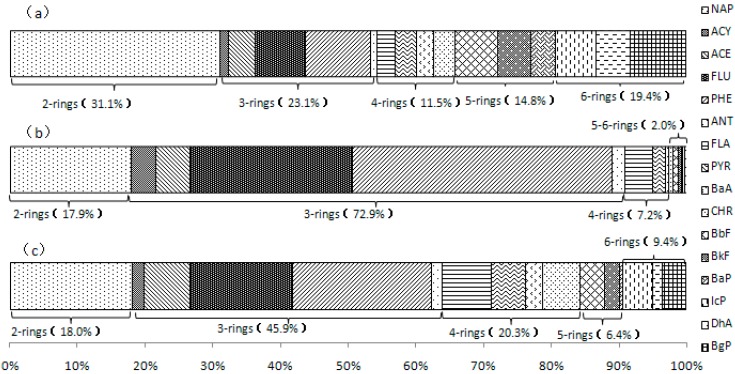
Mean concentration of individual PAHs in (**a**) WDP, (**b**) SPM and (**c**) sediment.

The concentration of 16 PAHs, summed as ∑PAHs, determined in WDP, are shown in Table 1. The concentrations of ∑PAHs ranged from 351 to 4427 ng/L with a mean value of 835 ng/L. Samples with measured low concentration for one compound may have high concentrations for other compounds. The average concentrations were 189, 140, 70.1, 89.9 and 118 ng/L for 2–6-ring PAHs, respectively. The compositional profiles of PAH in the dissolved phase are illustrated in Figure 2, which indicates that low molecular weight PAHs (2- and 3-ring PAHs) were abundant in samples, representing on average over 54% of all PAHs. For individual PAH, naphthalene and phenanthrene were dominant in water (Table 1). Their average concentrations were 189 and 57.8 ng/L, accounting for 31.1% and 9.5% of the average concentrations of ∑PAHs, respectively. In addition, the total concentration of potentially carcinogenic PAHs (∑PAH_7_) (BaA, CHR, BbF, BkF, BaP, IcP, DhA) ranged from 58.1 ng/L to 1344 ng/L with a mean value of 192 ng/L, accounting for 31.6% of ∑PAHs. The concentration of BaP was in the range of 5.02 to 240 ng/L with the mean value of 36.9 ng/L. The concentration of BaP in each water dissolved phase samples from the 37 sites was much higher than 2.8 ng/L (Environmental Quality Standard for surface Water of China, GB 3838-2002), suggesting water from Weihe River has a high health risk for drinking.

**Table 1:** Concentration ranges and mean value of polycyclic aromatic hydrocarbon (PAH) in water dissolved phase (WDP), suspended particulate matter (SPM) and sediment samples from Weihe River.

PAH	WDP (ng/L)		SPM (ng/L)		Sediment (ng/g dw)	
	Range	Mean	Range	Mean	Range	Mean
NAP	41.3–1002	189 ± 201	1024–26,598	3716 ± 1343	98.2–1605	360 ± 389
ACY	1.40–44.3	7.32 ± 5.40	89.0–4851	757 ± 235	7.01–117	37.5 ± 27.1
ACE	6.50–234	23.5 ± 19.5	213–7825	1056 ± 876	17.8–1247	134.7 ± 37.7
FLU	16.3–219	45.9 ± 15.2	706–35744	4974 ± 1786	29.2–1259	301 ± 182
PHE	21.4–208	57.8 ± 23.6	1021–58,048	7980 ± 2896	55.2–1998	414 ± 326
ANT	0.711–67.2	5.8 ± 1.9	43.0–2647	379 ± 122	4.94–474	31.6 ± 28.6
FLA	6.90–43.1	16.8 ± 6.9	110.2–6169	870 ± 213	15.0–1539	144 ± 122
PYR	6.32–48.6	18.5 ± 16.1	50.1–2976	406 ± 132	9.41–1146	99.4 ± 76.8
BaA	1.32–89.5	15.4 ± 12.2	2.1–773	85.5 ± 24.5	2.96–854	51.1 ± 35.8
CHR	1.51–122	19.4 ± 17.2	8.41–829	131 ± 36.3	3.81–1598	111 ± 89.7
BbF	6.40–245	38.4 ± 22.4	5.14–2262	167 ± 31.2	0.843–1057	72.2 ± 65.4
BkF	3.01–176	30.1 ± 19.3	3.30–773	104 ± 47.8	3.75–491	43.5 ± 34.5
BaP	5.02–240	36.9 ± 20.4	2.42–481	66.5 ± 36.8	6.80–1051	85.7 ± 45.8
IcP	3.34–252	30.6 ± 18.0	2.92–99.3	21.6 ± 8.91	1.90–342	29.8 ± 18.2
DhA	2.32–220	21.4 ± 11.4	0.315–147	21.2 ± 7.04	0.423–97.6	12.2 ± 9.91
BgP	3.94–411	51.1 ± 21.5	0.634–308	45.1 ± 18.2	2.02–794	72.6 ± 71.0
∑PAHs	351–4427	835	3557–147,907	20,780	362–15,667	2000

Compared with other polluted rivers in the world (Table 2), the mean concentrations of ∑PAHs in WDP from Weihe River were much higher than those found in Jinjiang River in China (53.2 ng/L) by Yang *et al.* [11], in the Susquehanna River in USA (67 ng/L) by Ko *et al.* [14], in the Yellow River Delta in China (121 ng/L) by Wang *et al.* [13] and in Henan Reach of Yellow River in China (662 ng/L) by Sun *et al.* [12]. It was even higher than those found in Sarno River (739 ng/L) (Italy) by Montuori and Triassi [5], which was defined as “the most polluted river in Europe”. It was obvious that PAHs concentrations in WDP from Weihe River were at a very high level compared with other rivers in the world.

**Table 2:** Concentration ranges and mean values of PAHs in WDP, SPM and sediment collected from different rivers in the world.

Medium	Locations	N ^a^	∑PAHs(ng/L, ng/g)		References
			Range	Mean	
WDP	Daliao River Estuary, China	16	139–1718	486	Men *et al*. [10]
	Yellow River Delta, China	16	64.8–335	121	Wang *et al*. [13]
	Jinjiang River, China	16	42.0–63.0	53.2	Yang *et al*. [11]
	Henan Reach of Yellow River, Middle China	16	144–2361	662	Sun *et al*. [12]
	Susquehanna River, USA	36	17–150	67	Ko *et al*. [14]
	Gaoping River, Taiwan, China	16	10–9400	430	Doong and Lin [15]
	Sarno River Estimate, Italy	16	12.4–2321	739	Montuori and Triassi [5]
	Weihe River, China	16	351–4427	835	This study
SPM	Daliao River Estuary, China	16	227–1405	467	Men *et al*. [10]
	Yellow River Delta, China	16	65.6–675	209	Wang *et al*. [13]
	Jinjiang River, China	16	144–217	174	Yang *et al*. [11]
	Sarno River Estimate, Italy	16	6.1–779	255	Montuori and Triassi [5]
	Henan Reach of Yellow River, Middle China	16	507–10,510	4101	Sun *et al*. [12]
	Tianjing River, China	16	938–64,200	8900	Shi *et al*. [4]
	Daliao River watershed, China	18	318–238,519	21,715	Guo *et al*. [16]
	Weihe River, China	16	3557–147,907	20,780	This study
Sediment	Guan River Estuary, China	19	90–218	133	He *et al*. [17]
	Tianjing River, China	16	787–1,943,000	10,980	Shi *et al*. [4]
	Pearl River, China	16	1434–10,811	4892	Mai *et al*. [18]
	Qiantang River, China	15	91–614	313	Chen *et al*. [9]
	Prai River, Malaysia	16	1102–7938	4357	Keshavarzifard *et al*. [6]
	Malacca River, Malaysia	16	716–1210	1023	Keshavarzifard *et al*. [6]
	Athabasca River, Canada	16	10–34,700		Headley *et al*. [2]
	Weihe River, China	16	362–15,667	2000	This study

Note: ^a^ N indicates the number of PAHs measured in the study.

### 3.2. PAHs Concentrations in SPM

As shown in Table 1, the concentrations of ∑PAHs in SPM samples range from 3557 ng/L in site 15 to 147,907 ng/L in site 34 with a mean value of 20,780 ng /L. In detail, they ranged from 1024 to 26,598 ng/L with a mean value of 3716 ng/L for 2-ring PAHs (NAP), from 2232 to 109,114 ng/L with a mean value of 15,146 ng/L for 3-ring PAHs (ACY, ACE, FLU, PHE, ANT), from 195 to 10,279 ng/L with a mean value of 1492 ng/L for 4-ring PAHs (FLA, PYR, BaA, CHR), from 12.1 to 3055 ng/L with a mean value of 292 ng/L for 5-ring PAHs (BbF, BkF, DhA) and from 8.01 to 763 ng/L with a mean value of 133 ng/L for 6-ring PAHs (BaP, BgP, ICP), respectively. They accounted for 17.9%, 72.9%, 7.2%, 1.4% and 0.6% of the ∑PAHs, respectively. Compared with the proportion of 2–6 ring PAHs in water dissolved phase, the proportion of 2-ring PAH decreased from 31.1% to 17.9%, while the proportion of 3-ring PAHs increased from 23.1% to 72.9%.

Compared with other polluted rivers in the world (Table 2), the ∑PAHs in SPM from Weihe River were much higher than most of other rivers. It was close to the Daliao River watershed (China) [10]. The level of PAHs in SPM of Weihe River was in the highest range.

### 3.3. PAHs Concentrations in Sediment

Table 1 illustrates the concentrations of PAHs in sediment samples in the study river. The concentrations of PAHs ranged from 362 to 15,667 ng/g dry weight (dw) with a mean value of 2000 ng/g. In terms of individual PAH composition, all of the 16 PAHs were detected at all sampling sites. The detected concentrations were 98.2–1605 ng/g dw for 2-ring PAHs, 201–5094 ng/g dw for 3-ring PAHs, 45.1–5137 ng/g dw for 4-ring PAHs, 4.92–1645 ng/g dw for 5-ring PAHs, and 16.1–2187 ng/g dw for 6-ring PAHs, respectively (Table 2). Two–six ring PAHs accounted for 18.0%, 45.9%, 20.3%, 6.4% and 9.4% of the concentration of PAHs, respectively (Figure 2). The concentration of potentially carcinogenic PAHs (∑PAH_7_) ranged from 34.4 to 5490 ng/g dw with the mean concentration of 406 ng/g dw, accounting for 20.2% of the total PAHs concentration. The concentration of BaP ranged from 6.80 to 1051 ng/g dw with a mean value of 85.7 ng/g dw.

Compared with other polluted rivers in the world (Table 2), the concentrations of ∑PAHs in sediment from Weihe River were much higher than those found in Guan River Estuary (China) by He *et al.* [17], Qiantang River (China) by Chen *et al.* [9] and Malacca River (Malaysia) by Keshavarzifard *et al.* [6]. Values higher than those found in Weihe River were found in Tianjing River (China) by Shi *et al.* [4], in Pearl River (China) by Mai *et al.* [18] and in Prai River (Malaysia) by Keshavarzifard *et al.* [6]. The concentrations of PAHs in sediment from Weihe River were in moderate range.

PAHs would rapidly become associated with SPMs and then aggregated in sediment after them into the aquatic environment. Therefore, relatively high concentration of PAHs in water and SPMs but moderate in sediment indicated that the contamination of PAHs in Weihe River might be caused by fresh input of PAHs.

## 4. Discussion

### 4.1. The Spatial Distribution of PAHs

The concentrations of ∑PAHs in WDPs, SPMs and sediments in the 37 sampling sites from Weihe River were illustrated in Figure 2. To WDP samples, the highest ∑PAHs were found in sites 22–28. The possible reason was that these sites were located in the mainstream and its branches of Weihe River in Xi’an district. Xi’an city is the most developed area in Weihe River catchment and a large quantity of PAHs entered the river in various ways. Especially the ∑PAHs of sample from site 24 was the highest among all the WDP samples. The possible cause was that site 24 located in Zaohe River, a branch of Weihe River, through which most of sewage from Xi’an area was discharged. The highest ∑PAHs in sediment samples was also found in this site.

**Figure 3:**
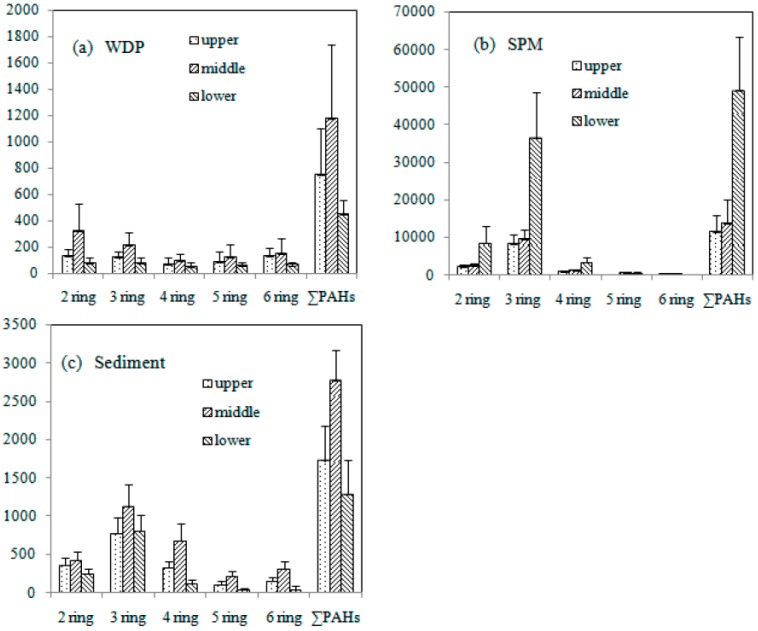
The concentration of PAHs in upper, middle and lower reaches in Weihe River.

The spatial distribution of PAHs in WDP, SPM and sediments were studied by comparing the concentrations of 2–6 rings PAHs among upper (sites 1–14), middle (sites 15–28) and lower (sites 29–37) reaches. The results were summarized in Figure 3. It could be observed from Figure 3a that both in WDP and sediment, the highest concentrations of ∑PAHs were observed in middle reach, while lowest concentrations of ∑PAHs were found in lower reach. For SPM, however, the highest concentrations were found in the lower reach and lowest concentrations were found in the upper reach. These results may be due to the following reasons. First, large quantities of waste water contained PAHs was discharged into middle reach of Weihe River, which caused the ∑PAHs in WDP were highest in middle reach. Second, the water in middle reach was flat and the SPM adsorbed PAHs from WDP, then sink to the bottom of the river, which caused the ∑PAHs in sediment increased. Third, the ∑PAHs in SPM were closely related to SPM content in water. The SPM contents in lower reach of Weihe River were very high (Table 3). The highest SPM contents were found in site 31, site 33 and site 34, which were high even to 24.4 g dw/L, 17.7 g dw/L and 31.6 g dw/L, respectively. The ∑PAHs in SPM from these three sites were also highest, which were 75,936 ng/L, 70,056 ng/L and 147,907 ng/L, respectively (Table 3). The lower reaches of Weihe River were characterized by torrential water flow with the high silt content, which caused that the PAHs in sediment were desorbed into water, and then be absorbed by SPM. Thus, ∑PAHs in sediments from lower reaches were even lower than those from upper reaches.

### 4.2. The Distribution of PAHs between WDP, SPM and Sediment

Many researchers and environmentalists considered that the environmental fate and behavior of hydrophobic organic compounds such as PAHs is ultimately determined by their physicochemical properties, such as organic content, size distribution, partition coefficient and salinity [19,20]. Physiochemical parameters such as COD (7.14–1056 mg/L), EC (546–1591 us/cm), TDS (154–1370 mg/L), salinity (0.27–0.63 ppt) and pH (7.46–9.96) in water samples from Weihe River were detected, but no significant correlations were observed between concentrations of PAH and these physiochemical parameters. The TOC of sediments in Weihe River were also detected, which ranged from 0.22% to 3.2% (Table 3). Although some papers reported positive linear relations between PAH concentrations and the total organic carbon contents (TOC) in sediment [21,22], no linear relation have been found between each individual PAH concentrations and TOC in Weihe River’s sediment.

The sediments and WDP system, and the SPM and WDP system in Weihe River are undergoing dynamic sorption and desorption and possibly have not reached chemical equilibrium, but an evaluation of the distribution of PAHs between sediments and WDP, and the SPM and WDP, can still provide useful information to understand the transport and fates of PAHs. In order to reach this purpose, the apparent distribution coefficient (K_d_) of total PAHs, which is defined as the ratio of total PAH in sediment or SPM to that in WDP, was calculated (Table 3). No correlations existed between K_d_ (sediment) and sediment TOC values for the 16 PAHs in Weihe River, which was similar to some other water bodies in the world [3,9,22]. The possible reason was that the sorption of PAHs to sediments will be affected by both organic matter content and the inorganic matrix (e.g., clay minerals) when sediment TOC values were low. In every site, the K_d_ (SPM) was much higher than K_d_(sediment) (Table 3), which probably indicated the fresh PAHs input in Weihe River.

**Table 3:** The ∑PAHs in SPM, TOC in sediment, the K_d_(SPM) and K_d_(sediment) in sampling sites.

Site No.	∑PAHs in WDP	SPM				Sediment		
		C_SPM_ (g/L）	∑PAHs (ng/L) ^a^	∑PAHs (ng/g) ^b^	K_d_(SPM) (L/g)	∑PAHs (ng/g)	TOC (%)	K_d_(sediment) (L/g)
1	1081	0.013	8493	663,539	614	738	0.863	0.684
2	340	0.018	8347	453,638	1333	684	2.95	2.01
3	429	0.033	7067	212,852	496	1571	2.54	3.66
4	656	0.026	6422	247,015	377	1356	1.23	2.07
5	400	0.031	6655	211,951	529	2712	1.02	6.77
6	394	0.0064	5725	894,463	2272	1238	1.19	3.14
7	454	0.0084	13,154	1,565,948	3446	1284	0.834	2.83
8	487	0.81	14,920	18,452	37.9	2139	0.572	4.39
9	357	0.044	11,512	259,278	727	3037	1.23	8.51
10	370	0.027	16,538	612,527	1655	2414	1.82	6.52
11	392	0.029	12,830	445,500	1137	1897	3.22	4.84
12	1106	0.065	13,818	213,236	193	2306	2.87	2.08
13	434	0.033	13,890	415,855	958	1387	0.486	3.19
14	371	0.050	19,654	396,255	1067	1457	0.885	3.92
15	372	0.013	3557	269,449	725	1354	0.218	3.64
16	362	0.045	13,983	310,736	860	1809	2.03	5.00
17	313	0.054	13,376	245,886	786	1772	2.22	5.67
18	335	0.024	12,664	523,323	1563	1569	2.70	4.69
19	298	0.18	13,306	75,175	253	1427	1.43	4.79
20	299	0.017	14,687	853,917	2860	1532	2.57	5.13
21	499	0.058	11,566	198,049	397	1481	0.709	2.97
22	1067	0.022	17,897	798,991	749	3010	0.937	2.82
23	1367	0.12	12,184	104,677	76.6	944	1.26	0.691
24	3486	0.65	18,936	29,204	8.38	15,667	1.83	4.49
25	825	0.034	9610	281,003	340	2999	2.11	3.63
26	1009	0.019	17,965	965,900	957			
27	1573	1.79	14,429	8067	5.13	1068	0.499	0.679
28	502	0.72	17,132	23,663	47.1	1331	0.324	2.65
29	264	7.93	25,833	3259	12.4	1334	0.514	5.05
30	245	0.0066	15,750	2,386,335	9747	1809	1.79	7.39
31	284	24.4	75,936	3113	11.0	1385	0.952	4.89
32	296	0.020	13,542	683,920	2314	1642	0.834	5.56
33	277	17.7	70,056	3953	14.2	1380	0.499	4.98
34	377	31.6	147,907	4687	12.4	1135	0.352	3.01
35	493	6.10	27,461	4501	9.13	1183	0.708	2.40
36	300	2.19	16,522	7544	25.2	392	0.558	1.31
37	345	6.09	25,516	4191	12.2	1564	0.61	4.54

Notes: ∑PAHs (ng/L) ^a^ = ∑PAHs in SPM per liter water; ∑PAHs (ng/g) ^b^ = ∑PAHs per gram SPM.

### 4.3. Source of PAHs in Weihe River

Pyrolytic and petrogenic sources are two major origins of anthropogenic PAHs in the environment. Understanding of the sources of PAHs is very important to study the transportation and fate of PAHs in environment. The molecular ratios of specific hydrocarbons were the most widely used method to distinguish the sources of PAHs in sediment environment. Ratios such as NAP/FLU, PHE/ANT, FLA/PYR, CHR/BaA, PYR/BaP, BaP/BeP, and MPHE/PHE have been developed for interpreting PAH composition and inferring the possible sources in many researches [13,23,24,25,26]. Among these molecular ratios, ratios of mass 178 and 202, were more widely used. For mass 178, ANT and PHE are two structural isomers, PHE is more thermodynamically stable. An ANT to ANT plus PHE (ANT/178) ratio < 0.1 usually is taken as an indication of petroleum while a ratio > 0.1 indicates a dominance of combustion. As for mass 202, a FLA to FLA plus PYR (FLA/(FLA + PYR)) ratio of 0.50 is usually defined as the petroleum/combustion transition point. A ration of FLA/(FLA + PYR) < 0.5 indicates a petrogenic source, while FLA/(FLA + PYR) > 0.5 means a pyrolytic origin.

**Figure 4:**
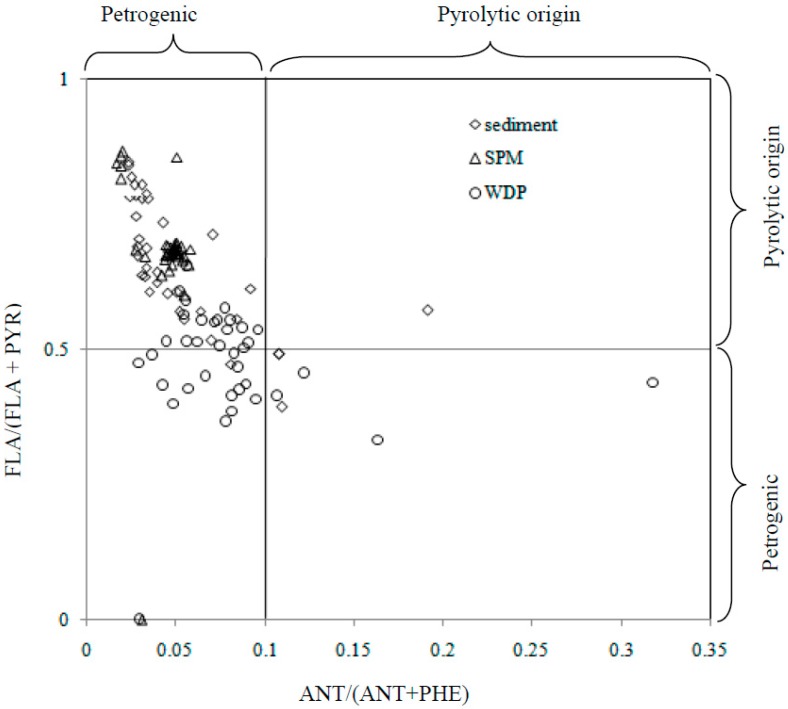
The ratios for ANT/(ANT + PHE) *versus* FLA/(FLA + PYR) in WDP, SPMs and sediment from Weihe River.

In this study, concentration ratios of FLA/(FLA + PYR) and ANT/(ANT + PHE) were used to distinguish the possible PAH origins in sediment in Weihe River. The ratios for ANT/(ANT + PHE) *versus* FLA/(FLA + PYR) were showed in Figure 4. It could be seen from Figure 4 that sediments from Weihe River originated from both pyrolytic and petrogenic sources. The pyrolytic source could be explained that coal burning was the main source for energy in this area. The petrogenic source was due to that many petroleum industry and chemical plant located in the drainage basin. Therefore, complex sources of PAHs contributed to the ∑PAHs burden in catchment of Weihe River.

### 4.4. Toxicity and Risk Assessment

Sediment Quality Guidelines (SQGs) on the basis of Biological Effects Database for Sediments (BEDS) have been suggested to evaluate the contamination effects in sediments of marine and estuarine ecosystems [27,28]. In this study, we applied four reliable sets of SQGs, including Effects Range-Low value (ERL), Effects Range-Median value (ERM), Probable Effects Level (PEL), and Threshold Effects Level (TEL), to evaluate the potential eco-toxicological risks of the individual and total PAH levels in the sediments of Weihe Rivers. Harmful biological impairments occur rarely (<ERL and <TEL), occasionally (≥ERL, ≥TEL, <ERM, and <PEL), and frequently (≥ERM and ≥PEL) based on the SQGs [27,28]. The concentrations of PAHs in Weihe River were compared with the ERL, ERM, PEL, and TEL values (Table 4).

**Table 4:** ERL ^a^, ERM ^b^, TEL ^c^ and PEL ^d^ guideline values for polycyclic aromatic hydrocarbons (ng/g dw).

PAHs	Upper	Middle	Lower	ERL	ERM	TEL	PEL
NAP	362.3	423.4	254.2	160	2100	34.6	391
ACY	33.2	39.9	38.7	44	640	5.87	128
ACE	94.7	190.2	118.2	16	500	6.71	88.9
FLU	270.7	345.5	273.7	19	540	21.2	144
PHE	358.4	499.7	362.9	240	1500	86.7	544
ANT	18.5	57.6	14.6	85	1100	46.9	245
FLA	116.0	227.7	64.3	600	5100	113	1494
PYR	91.8	156.2	28.3	665	2600	153	1398
BaA	34.4	97.4	10.2	261	1600	74.8	693
CHR	89.1	193.8	27.2	384	2800	108	846
BbF	44.4	137.6	22.6	–	–	–	–
BkF	47.2	62.1	10.7	–	–	–	–
BaP	12.1	16.6	5.9	430	1600	88.8	763
IcP	73.3	142.7	23.7	–	–	–	–
DhA	23.4	51.1	9.6	63.4	260	6.22	135
BgP	60.5	124.7	17.8	–	–	–	–
∑PAHs	1730	2766	1283	4022	44,792	1684	16,770

Notes: ^a^ ERL = effects range-low value [27]; ^b^ ERM = effects range-median value [27]; ^c^ TEL = threshold effect levels [28]; ^d^ PEL = probable effect levels [28].

The comparison of the mean values of individual PAH and total PAHs in the upper sediments of the Weihe River with the SQGs (Table 4) indicate that the concentrations of the NAP, ACE, FLU, PHE, FLA and total PAHs were higher than ERL values. The mean values of ACY and DhA were higher than TEL values. These results suggest the occurrence of occasional harmful biological impairments at upper reach of Weihe River. The mean values of ACE and FLU were even higher than corresponding PEL values, suggesting frequently harmful biological impairments.

The comparison of the mean values of individual PAH and total PAHs in the middle sediments of the Weihe River with the SQGs (Table 4) indicate all of the concentrations of individual PAH and total PAHs were higher than corresponding ERL or TEL values. These results suggest the occurrence of occasional harmful biological impairments at middle reach of Weihe River. The mean values of NAP, ACE and FLU were even higher than corresponding PEL values, suggesting frequently harmful biological impairments in middle reach in Weihe River.

The concentrations of PAH in lower reach of Weihe River were lowest. However, the concentrations of the NAP, ACY, ACE, FLU and PHE, were higher than ERL or TEL values, suggesting the occurrence of occasional harmful biological impairments. The ACE and FLU average concentrations were higher than corresponding PEL values. These results indicated harmful biological impairments occur frequently.

## 5. Conclusions

Analyses of Weihe River have provided very useful information for the evaluation of trace PAHs and probable sources in rivers that are located in the arid and semi-arid regions in Northwest China. The results obtained in this study showed that concentrations of total and individual PAHs in WDP, SPM and sediment varied significantly among sampling locations. The concentrations of PAH in Weihe River were at a higher level in comparison with other rivers worldwide. In both WDP and sediment, the highest concentrations of ∑PAHs were observed in middle reach, while lowest concentrations of ∑PAHs were found in lower reach. For SPM, however, the highest concentrations were found in lower reach and lowest concentrations were found in upper reach. The calculation of molecular ratio of ANT/(ANT + PHE) and FLA/(FLA + PYR) suggested a mixture of pyrolytic and petrogenic contamination in this river. The ecotoxicological risk evaluation based on international SQGs (ERL, ERM, TEL and PEL) indicated that harmful biological impairments would be caused by PAH compounds occasionally or frequently. It could be concluded that government officials should not only emphasize GDP growth, but also attach importance to environmental protection, and for environmental engineers should develop theories and devices that could fight environmental problems.

## References

[1] IARC (1987). IARC Monographs on the Evaluation of the Carcinogenic Risk of Chemicals to Humans.

[2] Headley J.V., Akre C., Conly F.M., Peru K.M., Dickson L.C. (2001). Preliminary characterization and source assessment of PAHs in tributary sediments of the Athabasca River, Canada. Environ. Forensics.

[3] Chen B., Xuan X., Zhu L., Wang J., Gao Y., Yang K., Shen X., Lou B. (2004). Distribution of polycyclic aromatic hydrocarbons in surface waters, sediments and soils of Hangzhou City, China. Water Res..

[4] Shi Z., Tao S., Pan B., Fan W., He X.C., Zuo Q., Wu S.P., Li B.G., Cao J., Liu W.X. (2005). Contamination of rivers in Tianjin, China by polycyclic aromatic hydrocarbons. Environ. Pollut..

[5] Montuori P., Triassi M. (2012). Polycyclic aromatic hydrocarbons loads into the Mediterranean Sea: Estimate of Sarno River inputs. Mar. Pollut. Bull..

[6] Keshavarzifard M., Zakaria M.P., Hwai T.S., Yusuff F.M., Mustafa S., Vaezzadeh V., Magam S.M., Masood N., Alkhadher S.A.A., Fatemeh A.-J. (2014). Baseline distributions and sources of polycyclic aromatic hydrocarbons (PAHs) in the surface sediments from the Prai and Malacca Rivers, Peninsular Malaysia. Mar. Pollut. Bull..

[7] Wang J.-Z., Chen T.-H., Zhu C.-Z., Peng S.-C. (2014). Trace organic pollutants in sediments from Huaihe River, China: Evaluation of sources and ecological risk. J. Hydrol..

[8] Dong J., Xia X., Wang M., Lai Y., Zhao P., Dong H., Zhao Y., Wen J. (2015). Effect of water–sediment regulation of the Xiaolangdi Reservoir on the concentrations, bioavailability, and fluxes of PAHs in the middle and lower reaches of the Yellow River. J. Hydrol..

[9] Chen Y., Zhu L., Zhou R. (2007). Characterization and distribution of polycyclic aromatic hydrocarbon in surface water and sediment from Qiantang River, China. J. Hazard. Mater..

[10] Men B., He M., Tan L., Lin C., Quan X. (2009). Distributions of polycyclic aromatic hydrocarbons in the Daliao River Estuary of Liaodong Bay, Bohai Sea (China). Mar. Pollut. Bull..

[11] Yang D., Qi S., Zhang Y., Xing X., Liu H., Qu C., Liu J., Li F. (2013). Levels, sources and potential risks of polycyclic aromatic hydrocarbons (PAHs) in multimedia environment along the Jinjiang River mainstream to Quanzhou Bay, China. Mar. Pollut. Bull..

[12] Sun J.-H., Wang G.-L., Chai Y., Zhang G., Li J., Feng J. (2009). Distribution of polycyclic aromatic hydrocarbons (PAHs) in Henan Reach of the Yellow River, Middle China. Ecotox. Environ. Safe..

[13] Wang L.L., Yang Z.F., Niu J.F., Wang J.Y. (2009). Characterization, ecological risk assessment and source diagnostics of polycyclic aromatic hydrocarbons in water column of the Yellow River Delta, one of the most plenty biodiversity zones in the world. J. Hazard. Mater..

[14] Ko F.C., Baker J., Fang M.D., Lee C.L. (2007). Composition and distribution of polycyclic aromatic hydrocarbons in the surface sediments from the Susquehanna River. Chemosphere.

[15] Doong R.A., Lin Y.T. (2004). Characterization and distribution of polycyclic aromatic hydrocarbon contaminations in surface sediments and water from Gao-ping River, Taiwan. Water Res..

[16] Guo W., He M., Yang Z., Lin C., Quan X., Wang H. (2007). Distribution of polycyclic aromatic hydrocarbons in water, suspended particulate matter and sediment from Daliao River watershed, China. Chemosphere.

[17] He X., Pang Y., Song X., Chen B., Feng Z., Ma Y. (2014). Distribution, sources and ecological risk assessment of PAHs in surface sediments from Guan River Estuary, China. Mar. Pollut. Bull..

[18] Mai B.X., Fu H.M., Sheng G.Y., Kang Y.H., Lin Z., Zhang G., Min Y.S., Zeng E.Y. (2002). Chlorinated and polycyclic aromatic hydrocarbons in riverine and estuarine sediments from Pearl River Delta, China. Environ. Pollut..

[19] Doong R.A., Peng C.K., Sun Y.C., Liao P.L. (2002). Composition and distribution of organchlorine pesticide residues in surface sediments from Wu-shi River Estuary, Taiwan. Mar. Pollut. Bull..

[20] Barker J.E., Eisenreich S.J., Eadie B.J. (1991). Sediment trap fluxes and benthic recycling of organic carbon, polycyclic aromatic hydrocarbons and polychlorobiphenyl congeners in Lake Superior. Environ. Sci. Technol..

[21] Witt G. (1995). Polyclclic aromatic hydrocarbons in water and sediment of the Balic Sea (Alaska). Mar. Pollut. Bull..

[22] Zhou J.L., Fileman T.W., Evans S., Donkin P., Readman J.W., Mantoura R.F.C., Rowland S.L. (1999). The partition of fluoranthene and pyrene between suspended particles and dissolved phased in the Humber Estuary: A study of the controlling factors. Sci. Total Environ..

[23] Yunker M.B., Snoedon L.R., Macdoland R.W., Smith J.N., Fowler M.G., Malaughlin F.A., Danyushevskaya A.I., Petrova V.I., Ivanov G.I. (1996). Polycyclic aromatic hydrocarbon composition and potential sources for sediment samples from the Beaufort and Barents Seas. Environ. Sci. Technol..

[24] Dickhut R.M., Canuel E.A., Gustafson K.E., Liu K., Arzayus K.M., Walker S.E., Edgecombe G., Gaylor M.O., MacDonald E.H. (2000). Automotive sources of carcinogenic polycyclic aromatic hydrocarbons associated with particular matter in the Chesapeake Bay region. Environ. Sci. Technol..

[25] Yim U.H., Hong S.H., Shim W.J., Oh J.R., Chang M. (2005). Spatio–temporal distribution and characteristics of PAHs in sediments from Masan Bay, Korea. Mar. Pollut. Bull..

[26] Li H., Lu L., Huang W., Yang J., Ran Y. (2014). *In situ* partitioning and bioconcentration of polycyclic aromatic hydrocarbons among water, suspended particulate matter, and fish in the Dongjiang and Pearl Rivers and the Pearl River Estuary, China. Mar. Pollut. Bull..

[27] Long E.R., Macdonald D.D., Smith S.L., Calder F.D. (1995). Incidence of adverse biological effects within ranges of chemical concentrations in marine and estuarine sediments. Environ. Manage..

[28] Macdonald D.D., Carr R.S., Calder F.D., Long E.R., Ingersoll C.G. (1996). Development and evaluation of sediment quality guidelines for Florida coastal waters. Ecotoxicology.

